# Anomalous right coronary artery originating from the aorta: a series of nine pediatric cases

**DOI:** 10.1186/s12887-023-04377-4

**Published:** 2023-10-31

**Authors:** Jia Na, Xi Chen, Zhen Zhen, Lu Gao, Yue Yuan

**Affiliations:** Department of Cardiology, Beijing Children’s Hospital Capital Medical University, National Center for Children’s Health, Beijing, 100045 China

**Keywords:** Anomalous origin of coronary artery, Pediatric, Clinical manifestations, Prognosis, Whole-exome sequencing, Case series

## Abstract

**Background:**

To investigate the clinical manifestations, prognosis, and possibly related genes of anomalous right coronary artery originating from the aorta (ARCA-L) in children.

**Methods:**

This case series study included pediatric patients diagnosed with ARCA-L at the Department of Cardiology in Beijing Children’s Hospital affiliated to Capital Medical University, between January 2017 and December 2019.

**Results:**

Nine pediatric patients (aged 3 months to 12 years, 4 boys) were included. Two cases presented with cardiac insufficiency as their primary manifestation, while the remaining seven had post-infection or post-exercise symptoms such as chest pain, chest tightness, long exhalation, lack of strength, and dizziness. Six patients displayed varying degrees of ST-T changes on the electrocardiograph, while two patients had a reduced left ventricular ejection fraction (LVEF) of 20-32% according to echocardiography. Multislice computed tomographic angiography confirmed the presence of ARCA-L in all patients. One patient underwent the unroofing technique. The remaining eight received conservative treatment. After a follow-up of 2–64 months, eight children had a good prognosis and survived. One child experienced sudden death due to aggravated heart failure. Whole exome sequencing revealed that one child tested negative, one had mutations in the RYR2 and LDB3 genes, and the remaining four patients had a mutation in the GDF1, LRP6, MEF2A, and KALRN genes, respectively.

**Conclusions:**

ARCA-L in children might have a wide variation in clinical manifestations and a risk of sudden death. The occurrence of the disease might be associated with genetic defects.

## Background

Anomalous aortic origin of a coronary artery (AAOCA) is a rare congenital cardiovascular disease with a prevalence of 0.1–1.15% [1]. In AAOCA, the two coronary arteries originate from the same aortic sinus, either through one orifice or two separate orifices. AAOCA cases are divided into anomalous left coronary artery from the right sinus (ALCA-R) and anomalous right coronary artery from the left sinus (ARCA-L). Coronary arteries with abnormal origins can be divided into five subtypes: pre-pulmonic, interarterial, subpulmonic, retroaortic, and retrocardiac [2].

AAOCA can be asymptomatic or have manifestations like angina-like chest pain [3]. Due to the lack of specificity of its clinical manifestations, it is easily misdiagnosed and missed diagnosis [4, 5]. Many children will be undiagnosed and participate in sports, and AAOCA is the second cause of sudden cardiac death (SCD) in young athletes in the United States of America and post-exertional syncope and SCD in adolescents [6–8]. The incidence of cardiac arrest in individuals < 21 years of age is 8 per 100,000 person-year [9]. Interarterial ARCA-L is 3–6 times more common than ALCA-R, and the occurrence of SCD is higher in patients with ALCA-R compared with ARCA-L [1]. Due to the rarity of AAOCA and ARCA-L and the different anomalies involved, the mechanism leading to SCD is unknown [3]. The postulated mechanisms include the presence of coronary ostial abnormalities, compression of an intramural segment during exercise, compression of an interarterial segment between the great vessels, and obstruction by a flap-like ridge related to an acutely angulated coronary artery, all contributing to myocardial ischemia and ventricular arrhythmia during physical exertion [10, 11]. A familial clustering of AAOCA was reported in 21% of the patients [12], but the potentially involved genes remain poorly known. ARCA-L can be managed surgically in selected cases [3]. Therefore, this study aimed to investigate the clinical manifestations, prognosis, and possibly related genes of pediatric ARCA-L.

## Methods

### Study design and patients

This case series included pediatric patients admitted to the Department of Cardiology in Beijing Children’s Hospital affiliated to Capital Medical University and diagnosed with ARCA-L between January 2017 and December 2019. The inclusion criteria were 1) < 18 years of age and 2) diagnosis of ARCA-L by multislice computed tomographic angiography (MSCTA). The exclusion criteria were (1) complex congenital heart disease, (2) other anomalous origin diseases of the coronary arteries, (3) acquired coronary artery lesions, or (4) incomplete data. The Ethics Committee of Beijing Children’s Hospital, affiliated to Capital Medical University, approved the study, including the data collection and analysis processes ([2023]-E-051-R). The requirement for individual consent was waived by the Ethics Committee of Beijing Children’s Hospital, affiliated to Capital Medical University, due to the retrospective nature of the study. I confirm that all methods were performed in accordance with the relevant guidelines. All procedures were performed in accordance with the ethical standards laid down in the 1964 Declaration of Helsinki and its later amendments. All data were anonymized after extraction and before analysis. The ethical committee has the responsibility to ensure that the data is properly handled and that confidentiality is respected.

### Data collection

All included cases had data from electrocardiogram (ECG), echocardiography, and MSCTA. The diagnostic criteria for ARCA-L were MSCTA examination or surgical confirmation. The American College of Cardiology/American Heart Association (ACC/AHA) management guidelines for congenital heart disease in adults propose a computed tomographic angiography (CTA) examination as the recommended class I option for the ectopic origin of coronary arteries [13]. The MSCTA diagnostic criteria were the origin of the right coronary artery from the left coronary sinus. The images of the coronary arteries, aorta, and pulmonary arteries were reconstructed using a Revolution 256-row spiral CT (GE Healthcare, Waukesha, WI, USA).

A Philips IE33 color Doppler cardio-diagnostic apparatus was used to explore the heart’s structure, the location of the coronary artery openings, their branching pathways, and the cardiac contractile function. The coronary artery opening and its branching path were identified through the root section of the thoracic anterior short-axis large vessels. The color Doppler velocity scale was set at 30–50 cm/s to obtain the coronary arterial blood flow signal. A left ventricular ejection fraction (LVEF) < 56% was defined as decreased cardiac function. After consent from the legal guardians, 2 mL of peripheral blood was drawn from the patients and their parents for whole exome sequencing (WES).

The ACC/AHA guidelines [2] recommend surgical intervention in patients with ARCA-L with evidence of myocardial ischemia or ventricular arrhythmias, with recommendation classes I and IIa, respectively. Surgical intervention (IIb) or continuous observation (IIb) is feasible in patients without the above complications. Surgical methods include the unroofing technique of the coronary artery and the grafting of the coronary artery. Close follow-up is recommended for non-surgical cases, and medical therapy includes β-blockers and avoiding physical over-exertion.

All patients were followed up at the outpatient clinic and by telephone until March 2023. Data were extracted from the medical records of the patients, including sex, age of onset, age of diagnosis, clinical manifestations, electrocardiogram, imaging examinations, therapy, and prognosis. The typical ECG features of right coronary artery occlusion are ST-segment elevation seen in leads II, III, and aVF, and the evolution of T waves from low to high and then inverted, which may show necrotic Q waves.

### Statistical analysis

Only descriptive analysis was performed. The categorical data were expressed as n (%). The continuous data were expressed as ranges.

## Results

Nine pediatric patients were included. There were four males. They were aged from 3 months to 12.75 years (median age of 10.08 years). Seven patients had non-specific symptoms such as chest pain, chest tightness, long expiration, lack of strength, and dizziness as the main clinical manifestations, mostly after an infection or exercise. Two children had cardiac insufficiency-related symptoms, including one 3-month-old infant who manifested crying, dysphoria, cyanosis, and oliguria, and one 2-year-old child whose main manifestation was obvious intolerance to daily activities.

**Fig. 1:**
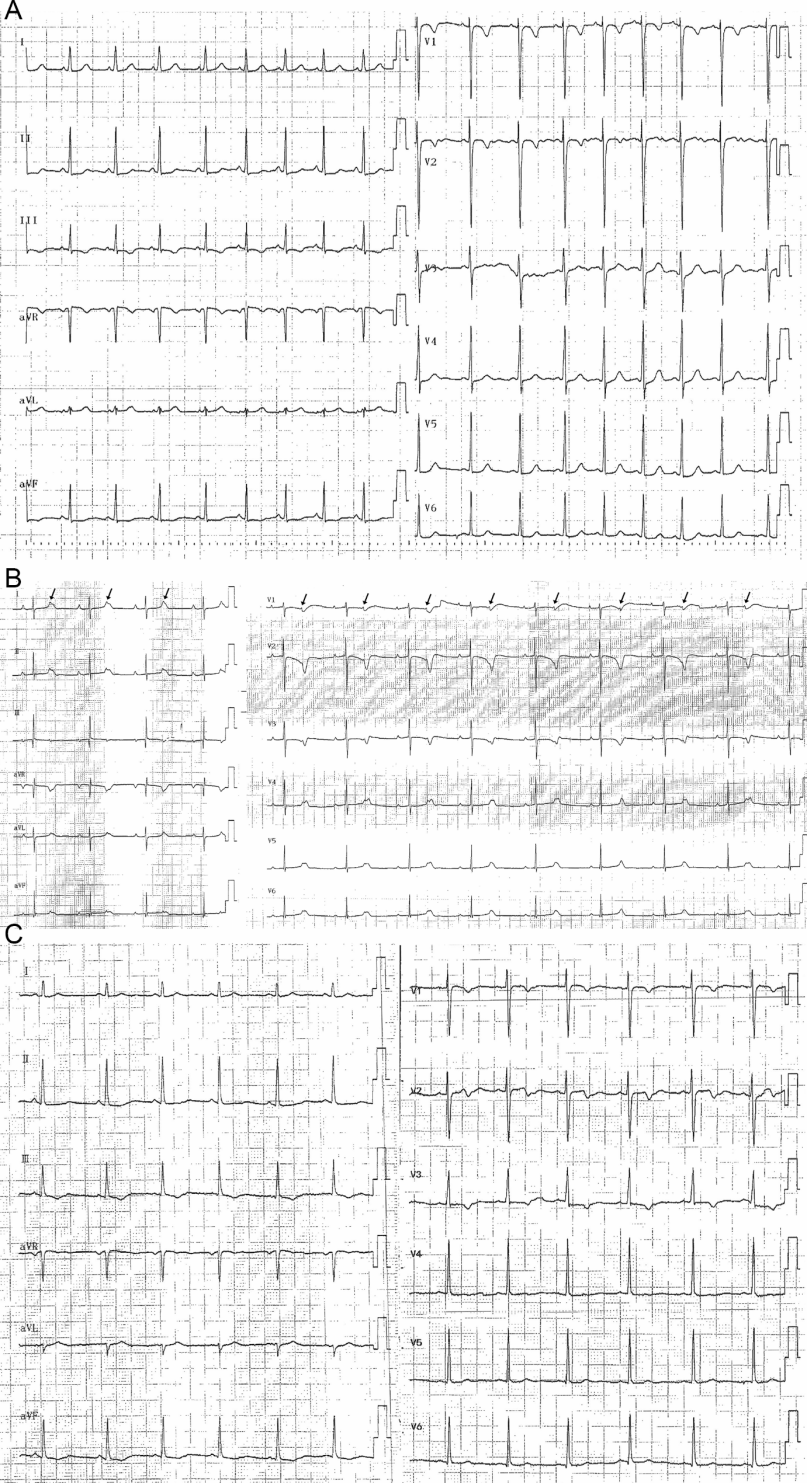
(**A**) ECG of case #2 showing I, II, III, aVF, and V4-V6 lead ST-segment downshifts, III lead T-wave inversion, and aVF lead bidirectional T-wave. (**B**) ECG of case #3 showing second-degree atrioventricular block, with a fixed conduction ratio of 2:1. The arrows indicate the P-wave with conduction block. (**C**) ECG of case #8 showing II, III, aVF, and V3-V6 lead T-wave changes

**Fig. 2:**
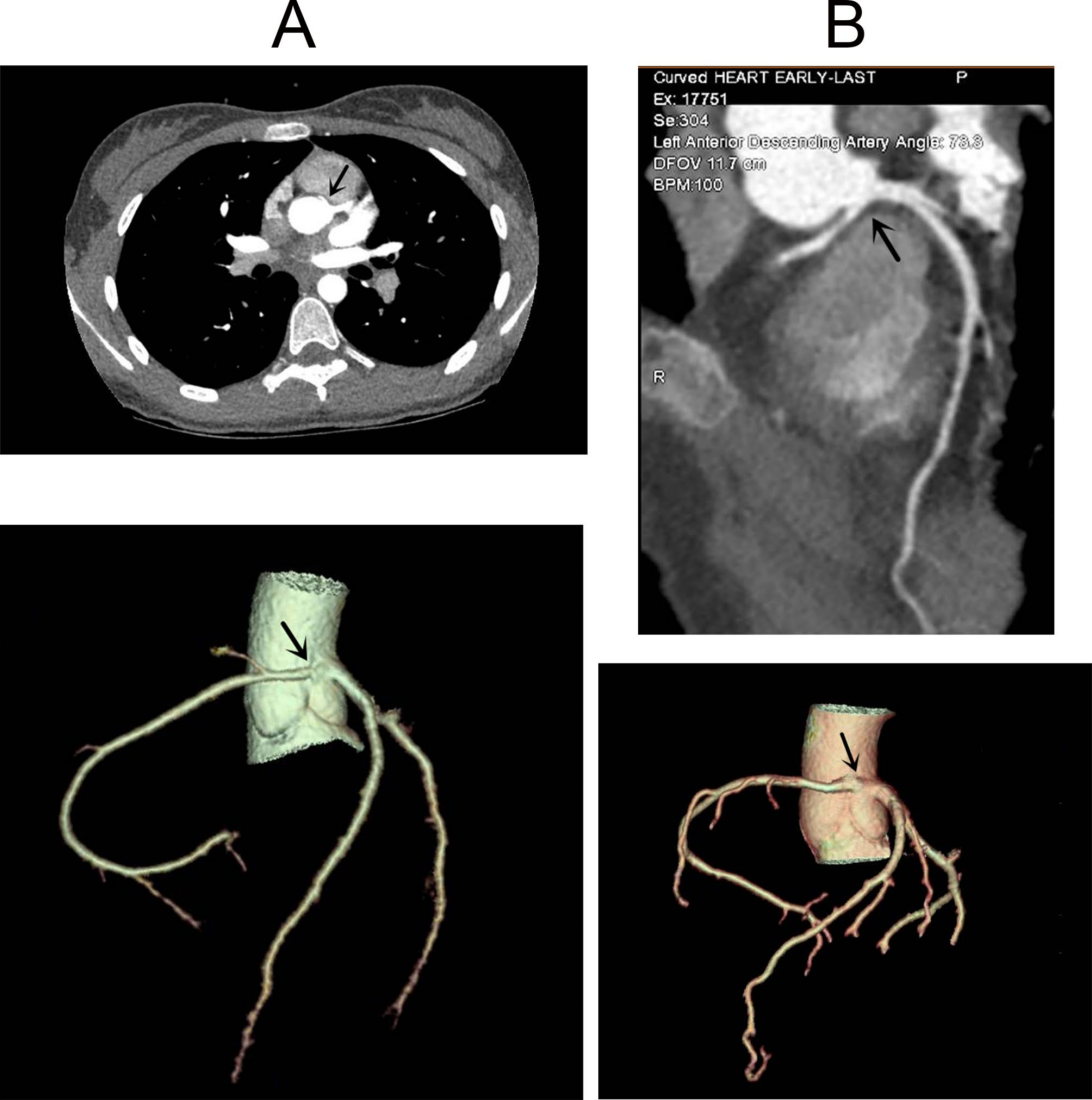
(**A**) MSCTA of case #8 showing the origin of the right coronary artery from the left coronary sinus. (**B**) MSCTA of case #9 showing the origin of the right coronary artery from the left coronary sinus and traveled between the aorta and pulmonary arteries

Non-specific ST-T changes were the main ECG manifestation in six patients. An intraventricular block was found on ECG in one patient. Second-degree sinoatrial block was found in three patients by Holter monitor. Second-degree atrioventricular block was found in two patients by ECG and Holter monitor (Fig. 1). The echocardiography in seven patients showed no significant abnormalities in each atrial internal diameter and cardiac function. The echocardiography of two patients showed severe enlargement of the left ventricular internal diameter, and LVEF was 20-32%. The echocardiography of one patient suggested a possibility of abnormal coronary artery origin. One patient underwent cardiac magnetic resonance, which was normal. MSCTA showed ARCA-L in all nine patients, and six patients had an origin between the aorta and the pulmonary artery (Fig. 2). WES was conducted on six children, revealing that one child tested negative, one had mutations in the RYR2 and LDB3 genes, while the remaining four patients had a mutation in the GDF1, LRP6, MEF2A, and KALRN genes, respectively (Table 1).

**Table 1:** Whole-exon sequencing

Case #	Gene	Nucleotide/amino acid changes	Homozygote/ Heterozygote	Normal human frequency	Projections	Pathogenicity analysis	Mode of inheritance	Disease/Phenotype
1	RYR2	c.6593G > A (p.R219 8 H) exon43	Heterozygote	0.00008	Benign	Uncertain	1. Autosomal dominant 2. Autosomal dominant	1. Catecholamine-sensitive polymorphic ventricular tachycardia type 1 2. Arrhythmogenic right ventricular dysplasia/ cardiomyopathy type 2
	LDB3	c.944 C > T (p.P315L) exon7	Heterozygote	0.0001	Benign	Uncertain	1. Autosomal dominant 2. Autosomal dominant	1. Dilated cardiomyopathy type 1 C, with or without LVNC 2. Myofibrillar myopathy, type 4
2	GDF1	c.503 C > T (p.A168V) exon8	Heterozygote		Benign	Uncertain	1. Autosomal recessive 2. Autosomal dominant	1. Right atrial isomerism 2. Dextral aortic ectopia type 3
4	LRP6	c.1276 C > T (p.R426X) exon6	Heterozygote			Likely pathogenic	Autosomal dominant	Coronary artery disease type 2
5	Negative							
7	MEF2A	c.743G > A (p.G248D) exon8	Heterozygote	0.0004	Benign	Uncertain	Autosomal dominant	Coronary artery disease type 1
8	KALRN	c.2707 C > T (p.L903L) exon16	Heterozygote	0.0092		Uncertain		Coronary heart disease susceptibility type 5

Seven children were treated orally with low doses of metoprolol, with a starting dose of 0.1–0.3 mg/kg/d and a maximum dose of 2 mg/kg/d. Two patients with combined decreased cardiac function were given oral digoxin 5–10 µg/kg/d and continuous intravenous pumping of milrinone 0.25-1.00 µg/kg/min, oral hydrochlorothiazide, spironolactone, and captopril. One patient (#6) had a proposed diagnosis of endocardial fibroelastosis before the completion of MSCTA and was given glucocorticoids. One patient (#9) underwent the unroofing technique of the coronary artery.

Patient #9 was followed up for 2 months after surgery, with no specific symptoms and an acceptable postoperative recovery. Seven patients (#1, 2, 3, 5, 6, 7, and 8) were followed up for 43–64 months, with cardiac symptoms, ECG, and cardiac function improvement after conservative medical treatment, without complaints of discomfort or adverse events, and with an acceptable quality of life. One patient (#4) experienced sudden death due to aggravated heart failure.

## Discussion

The results showed various manifestations of pediatric ARCA-L with a risk of sudden death. The occurrence of the disease might be associated with genetic defects. These findings might provide new insight for managing ARCA-L in terms of related genes.

AAOCA, including ARCA-L, is a potentially lethal condition associated with malignant arrhythmia, stenocardia, myocardial infarction, and SCD in young adults, highlighting the need to diagnose AAOCA and ARCA-L [5, 14]. D’Ascenzi et al. [15] reported that AAOCA accounts for 7.2% of the SCDs in athletes and 1.9% of the SCDs in non-athletes. ARCA-L accounts for approximately 0.02–0.2% of all anomalous origin of the coronary arteries [16]. Patients may manifest in clinical terms from asymptomatic to myocardial ischemic symptoms. This study suggested that the clinical manifestations in pediatric patients lack specificity. The symptoms are probably related to coronary artery compression, narrowing the lumen. The interarterial course coronary artery is at an acute angle with the aorta, which can lead to slit-like openings and open flap-like ridges, and aortectasia can obstruct the coronary artery with slit-like openings forming live valves, causing myocardial ischemia and fibrosis and increasing the chance of fatal arrhythmias and even SCD [17].

The most common ECG abnormalities in AAOCA patients are ST-T changes, suggesting the existence of myocardial ischemia in the corresponding donor area, followed by conduction block [18]. An abnormal ECG indicates a pathological process or heart anomaly, but it does not diagnose AAOCA by itself [3, 19]. Transthoracic echocardiography can identify the coronary artery opening and its course through the root section of the thoracic anterior short-axis large vessels [20]. Echocardiography was shown to increase the detection rate of AAOCAs [3, 21, 22], but ultrasound examinations are notoriously operator-dependent, and even ultrasound systems perform better than others. Nevertheless, examinations such as ECG and echocardiography also appear to have limited accuracy in diagnosing ARCA-L, with abnormal examinations in only some patients in the present study. Only one case (case #9) showed a possible abnormal origin of the right coronary artery on echocardiography, while the other eight cases did not. The ECG changes of the patients were mainly manifested by non-specific ST-T changes and no pathological Q-wave. Therefore, CTA might be a better examination to diagnose ARCA-L, and it was the only positive examination for all nine patients included in this study. Still, diagnostic value analyses of the diagnostic modalities were impossible due to the small sample size. In this study, segmental ventricular wall motion abnormalities under echocardiography could be highly suggestive of abnormalities related to the coronary artery blood supply and the need to identify coronary artery opening and pathway. Still, the sample size was too limited to perform diagnostic accuracy analysis.

In the present study, the diagnostic criterion for ARCA-L was a confirmation by MSCTA, and it remains unknown whether some patients who did not undergo MSCTA or with an apparently normal MSCTA were missed. Still, the anatomical basis for diagnosing ARCA-L is the anomalous origin of the proximal segment of the coronary artery, and MSCTA is an examination of choice for detecting such changes. MSCTA has become the predominant examination method for the diagnosis of abnormal coronary artery origin, which can not only clearly show the origin and path of the coronary artery from multiple angles but also measure the stenotic meridian and the angle between the proximal vessels and the aorta formation, and also observe the relationship between the aberrant coronary artery and the aorta, pulmonary artery, and surrounding tissues, and can provide important information for the making of surgical plans [23, 24]. Krishnamurthy et al. [25] showed that CTA was accurate in recognition of AAOCA in 100% of their patients.

The pathogenesis of AAOCA, including ARCA-L, is unknown, and the present study, being retrospective, could not provide any clue on the subject. More extensive research is needed to understand the underlying causes of the disease better. Kayalar et al. [26] suggested that dysplasia of one side of the primordium is the cause of unilateral coronary artery loss and that a caecus appears in the aortic sinus on the absent side, which is the coronary artery on the undeveloped side. In addition, the unbalanced development of the parts at the aortic sinus can cause translocation of the primordium of one side of the coronary artery or fusion with the primordium of the contralateral coronary artery [26]. AAOCA can be associated with congenital heart diseases such as arcus aortae atresia, perimembranous ventricular septal defect, or tetralogy of Fallot [27]. Studies have shown that congenital heart disease is associated with chromosomal abnormalities, monogenic gene defects, and polygene defects, and genetic loci found to be associated with the development of congenital heart disease include NKX2.5, GATA4, TBX1, TBX5, FOX1, Lefty, SMAD3, GDF1, HAND1, and HAND2 [28, 29]. Significant abnormalities in coronary artery morphology have been reported in GJA1 gene pure-hybrid knockout mice [30]. Some researchers have sequenced the GJA1 gene in patients with anomalous origin of the left coronary artery from the pulmonary artery, but no mutations were found [31]. No mutations or conduction pathway abnormalities associated with AAOCA have been reported in the literature. In the present study, six patients underwent WES during their clinical management. Patient #4 had a mutation in the LRP6 gene, a suspected pathogenic variant. Currently, the known clinical phenotype due to this mutation is coronary artery disease type 2 [32], which is characterized by reduced or absent blood flow in one or more coronary arteries and is consistent with the MSCTA phenotype in this pediatric patient. This patient had more serious clinical manifestations of cardiac insufficiency, and echocardiography showed severe impairment of cardiac structure and function. In one patient, mutations in RYR2 and LDB3 genes were found. The known diseases caused by mutations in the RYR2 gene are catecholamine-sensitive polymorphic ventricular tachycardia and arrhythmogenic right ventricular cardiomyopathy [33]. The known disease caused by mutations in the LDB3 gene is dilated cardiomyopathy [34]. One child was found to have a mutation in the GDF1 gene, and the known diseases caused by this mutation are right atrial heterogeneity and dextral aortic ectopia [35]. One patient was found to have a mutation in the KALRN gene and a known clinical phenotype of coronary heart disease susceptibility type 5 [36], which is characterized by an imbalance between the functional requirements of the myocardium and the coronary blood supply and the coronary artery insufficient blood supply might be related to the thickening and loss of elasticity of the coronary arteries. Still, additional large-scale studies are necessary to determine the contribution of genetics to ARCA-L. A previous study reported familial clustering of AAOCA in 21% of the patients [12]. In the present study, one patient was found to be without mutations.

The therapy of AAOCA, including ARCA-L, mainly includes follow-up observation, exercise restriction, drug therapy, and surgery [3]. Patients without evidence of myocardial ischemia can be treated with β-blockers, such as metoprolol, when there are no contraindications, which can control the ventricular rate, inhibit myocardial contraction, and reduce oxygen consumption [37]. Other drugs, including nitrate medications, dilate the coronary arteries and increase myocardial blood supply; non-dihydropyridine calcium antagonists can prevent and control coronary artery spasms. Seven patients in this group were given metoprolol orally, and one stopped metoprolol after surgery. The remaining six patients survived more than 3 years of follow-up without uncomfortable complaints or adverse events. The indications for surgical operations are not uniform. All patients < 30 years of age with an abnormal origin of the coronary arteries and with interarterial traveling segments should undergo surgery [3]. The ACC/AHA guidelines [2] recommend surgical intervention for ARCA-L with manifestations of myocardial ischemia or ventricular arrhythmias. Surgical intervention (class IIb) or continuous observation (class IIb) is feasible in patients without such symptoms [2]. Mosca et al. [38] also suggested that (1) surgical intervention should be performed in patients with symptomatic AAOCA, (2) asymptomatic patients with anomalous left coronary artery origin and older than 10 years should be treated with surgery, (3) asymptomatic patients with anomalous right coronary artery origin can be individualized treatment according to the patient’s needs and abnormal type, and (4) medical treatment is recommended for those older than 40–45 years of age, asymptomatic, and without evidence of myocardial ischemia. Surgical options include coronary artery bypass grafting, unroofing of the coronary artery, and replacement of the coronary artery ostium [39, 40].

Many patients with AAOCA have a favorable prognosis, but ARCA-L might have a poor prognosis, but there is still a lack of hard evidence about the prognosis of patients with untreated AAOCA [2]. The prognosis of a child with AAOCA or ARCA-L is closely related to the establishment of collateral circulation and the combination of other structural cardiac anomalies [3]. In the present study, one patient underwent surgery and was well during follow-up. Seven patients were managed conservatively, with different symptoms, all manageable and with good quality of life. One patient died suddenly.

This study had limitations. It was a single-center study over a relatively short period. It was a retrospective study with a small sample size and limited to the data available in the charts, which was only descriptive analysis could be performed. In addition, WES was performed in only six patients, limiting the available data. For example, more detailed cardiac examinations were unavailable, such as invasive coronary angiography, cardiac magnetic resonance, and nuclear scintigraphy. One patient in this study underwent surgery at another hospital. The invasive coronary angiography, cardiac MR, and nuclear scintigraphy data are unavailable. Multicenter studies could help address the sample size issue.

## Conclusions

In conclusion, ARCA-L in children has wide clinical manifestations and a risk of sudden death, which might be associated with gene mutations. Further studies with large sample sizes are needed to determine whether it is an unknown clinical phenotype of known causative genes.

## Data Availability

All data generated or analyzed during this study are included in this published article.

## List of abbreviations

ARCA-L: Anomalous right coronary artery originating from the aorta
LVEF: Left ventricular ejection fraction
AAOCA: Anomalous aortic origin of a coronary artery
ALCA-R: Anomalous left coronary artery from the right sinus
SCD: Sudden cardiac death
MSCTA: Multislice computed tomographic angiography
ECG: Electrocardiogram
ACC/AHA: American College of Cardiology/American Heart Association
CTA: Computed tomographic angiography
WES: Whole exome sequencing

## References

[1] Tessitore A, Caiffa T, Bobbo M, D’Agata Mottolese B, Barbi E, Chicco D (2022). Anomalous aortic origin of coronary artery: for a challenging diagnosis, a transthoracic echocardiogram is recommended. Acta Paediatr.

[2] Cheezum MK, Liberthson RR, Shah NR, Villines TC, O’Gara PT, Landzberg MJ, Blankstein R (2017). Anomalous aortic origin of a coronary artery from the Inappropriate Sinus of Valsalva. J Am Coll Cardiol.

[3] Molossi S, Martinez-Bravo LE, Mery CM (2019). Anomalous aortic origin of a coronary artery. Methodist Debakey Cardiovasc J.

[4] Brothers JA, Gaynor JW, Jacobs JP, Caldarone C, Jegatheeswaran A, Jacobs ML (2010). Anomalous coronary artery Working G: the registry of anomalous aortic origin of the coronary artery of the congenital heart surgeons’ Society. Cardiol Young.

[5] Molossi S, Agrawal H (2017). Coronary artery anomalies: a multidisciplinary approach to shape the landscape of a challenging problem. Congenit Heart Dis.

[6] Maron BJ, Doerer JJ, Haas TS, Tierney DM, Mueller FO (2009). Sudden deaths in young competitive athletes: analysis of 1866 deaths in the United States, 1980–2006. Circulation.

[7] Stecker EC, Reinier K, Marijon E, Narayanan K, Teodorescu C, Uy-Evanado A, Gunson K, Jui J, Chugh SS (2014). Public health burden of Sudden Cardiac Death in the United States. Circ Arrhythm Electrophysiol.

[8] Link MS, Estes NA (2010). Athletes and arrhythmias. J Cardiovasc Electrophysiol.

[9] Atkins DL, Everson-Stewart S, Sears GK, Daya M, Osmond MH, Warden CR, Berg RA (2009). Resuscitation outcomes Consortium I: epidemiology and outcomes from out-of-hospital Cardiac Arrest in children: the Resuscitation outcomes Consortium Epistry-Cardiac arrest. Circulation.

[10] Brothers J, Carter C, McBride M, Spray T, Paridon S (2010). Anomalous left coronary artery origin from the opposite sinus of Valsalva: evidence of intermittent ischemia. J Thorac Cardiovasc Surg.

[11] Cheitlin MD, MacGregor J (2009). Congenital anomalies of coronary arteries: role in the pathogenesis of Sudden Cardiac Death. Herz.

[12] Agrawal H, Mery CM, Sexson Tejtel SK, Fraser CD, McKenzie ED, Qureshi AM, Molossi S (2018). Familial clustering of cardiac conditions in patients with anomalous aortic origin of a coronary artery and myocardial bridges. Cardiol Young.

[13] Stout KK, Daniels CJ, Aboulhosn JA, Bozkurt B, Broberg CS, Colman JM, Crumb SR, Dearani JA, Fuller S, Gurvitz M (2019). 2018 AHA/ACC Guideline for the management of adults with congenital Heart Disease: a report of the American College of Cardiology/American Heart Association Task Force on Clinical Practice guidelines. Circulation.

[14] Shaban M, Budhathoki P, Bhatt T, Lee S, Urena Neme AP, Rodriguez Guerra MA, Zaw M (2022). Anomalous origin of the right coronary artery: an uncommon presentation. Cureus.

[15] D’Ascenzi F, Valentini F, Pistoresi S, Frascaro F, Piu P, Cavigli L, Valente S, Focardi M, Cameli M, Bonifazi M (2022). Causes of Sudden Cardiac Death in young athletes and non-athletes: systematic review and meta-analysis: Sudden Cardiac Death in the young. Trends Cardiovasc Med.

[16] Greet B, Quinones A, Srichai M, Bangalore S, Roswell R (2012). Anomalous right coronary artery and Sudden Cardiac Death. Circ Arrhythm Electrophysiol.

[17] Wang D, Wu Y, Liu Y (2017). A case of sudden death due to anomalous origins of the left coronary artery from the opposite sinus of Valsalva. J Cardiovasc Pulmonary Dis.

[18] Williams SB, Pham TDN, Doan TT, Reaves-O’Neal D, Bonilla-Ramirez C, Binsalamah ZM, Mery CM, Caldarone CA, Molossi S (2022). Pattern, behavior, and clinical implications of electrocardiographic changes in patients undergoing repair of anomalous aortic origin of coronary arteries. J Thorac Cardiovasc Surg.

[19] Gentile F, Castiglione V, De Caterina R (2021). Coronary artery anomalies. Circulation.

[20] Thankavel PP, Lemler MS, Ramaciotti C (2015). Utility and importance of new echocardiographic screening methods in diagnosis of anomalous coronary origins in the pediatric population: assessment of quality improvement. Pediatr Cardiol.

[21] Bianco F, Colaneri M, Bucciarelli V, Surace FC, Iezzi FV, Primavera M, Biasi A, Giusti G, Berton E, Baldoni M et al. Echocardiographic screening for the anomalous aortic origin of coronary arteries. Open Heart 2021, 8. 10.1136/openhrt-2020-001495 PMC7802674 33431619

[22] Cantinotti M, Giordano R, Assanta N, Koestenberger M, Franchi E, Marchese P, Clemente A, Kutty S, D’Ascenzi F. Echocardiographic screening of anomalous origin of coronary arteries in athletes with a focus on high take-off. Healthc (Basel) 2021, 9. 10.3390/healthcare9020231 PMC7924023 33672577

[23] Zhao J, Zhang Y, Li G (2018). Anomalous origin of right coronary artery from the left coronary sinus in children: a case report and literature. J Clin Pediatr.

[24] Cao A, Guo H, Wu L (2016). Value of dual-source CT coronary angiography to the diagnosis of coronary-pulmonary artery fistula. J Practical Diagnosis Therapy.

[25] Krishnamurthy R, Masand PM, Jadhav SP, Molossi S, Zhang W, Agrawal HM, Mery CM (2021). Accuracy of computed tomography angiography and structured reporting of high-risk morphology in anomalous aortic origin of coronary artery: comparison with Surgery. Pediatr Radiol.

[26] Kayalar N, Burkhart HM, Dearani JA, Cetta F, Schaff HV (2009). Congenital coronary anomalies and surgical treatment. Congenit Heart Dis.

[27] Zingarelli A, Seitun S, Boccalini S, Budaj I, Zawaideh C, Valbusa A, Balbi M, Bezante GP, Brunelli C (2015). Anomalous single coronary artery (R-type) in the elderly: description of benign and isolated variant. Int J Cardiol.

[28] Yasuhara J, Garg V (2021). Genetics of congenital Heart Disease: a narrative review of recent advances and clinical implications. Transl Pediatr.

[29] Fahed AC, Gelb BD, Seidman JG, Seidman CE (2013). Genetics of congenital Heart Disease: the glass half empty. Circ Res.

[30] Li WE, Waldo K, Linask KL, Chen T, Wessels A, Parmacek MS, Kirby ML, Lo CW (2002). An essential role for connexin43 gap junctions in mouse coronary artery development. Development.

[31] Sawaya F, Souki R, Arabi M, Majdalani M, Obeid M, Bitar FF, Nemer G (2011). Absence of GJA1 gene mutations in four patients with anomalous left coronary artery from the pulmonary artery (ALCAPA). J Med Liban.

[32] Guo Q, Lai Y, Chu J, Chen X, Gao M, Sang C, Dong J, Pu J, Ma C (2021). LRP6 polymorphisms is Associated with Sudden Cardiac Death in patients with Chronic Heart Failure in the Chinese Han Population. Front Cardiovasc Med.

[33] Perez-Riera AR, Barbosa-Barros R, de Rezende Barbosa MPC, Daminello-Raimundo R, de Lucca AA (2018). Jr., De Abreu LC: Catecholaminergic polymorphic ventricular tachycardia, an update. Ann Noninvasive Electrocardiol.

[34] Wang DF, Lyu JL, Fang J, Chen J, Chen WW, Huang JQ, Xia SD, Jin JM, Dong FH, Cheng HQ (2019). Impact of LDB3 gene polymorphisms on clinical presentation and implantable cardioverter defibrillator (ICD) implantation in Chinese patients with idiopathic dilated cardiomyopathy. J Zhejiang Univ Sci B.

[35] Zhang J, Wu Q, Wang L, Li X, Ma Y, Yao L (2015). Association of GDF1 rs4808863 with fetal congenital heart defects: a case-control study. BMJ Open.

[36] Wang L, Hauser ER, Shah SH, Pericak-Vance MA, Haynes C, Crosslin D, Harris M, Nelson S, Hale AB, Granger CB (2007). Peakwide mapping on chromosome 3q13 identifies the kalirin gene as a novel candidate gene for coronary artery Disease. Am J Hum Genet.

[37] Yildiz O, Karabay KO, Akman C, Aytekin V (2015). Anomalous origin of the left main coronary artery from the right coronary artery with a preaortic course. Tex Heart Inst J.

[38] Mosca RS, Phoon CK (2016). Anomalous aortic origin of a coronary artery is not always a Surgical Disease. Semin Thorac Cardiovasc Surg Pediatr Card Surg Annu.

[39] Jaggers J, Lodge AJ. Surgical therapy for anomalous aortic origin of the coronary arteries. Semin Thorac Cardiovasc Surg Pediatr Card Surg Annu 2005:122–7. 10.1053/j.pcsu.2005.01.004 15818367

[40] Jegatheeswaran A, Alsoufi B (2021). Anomalous aortic origin of a coronary artery: 2020 year in review. J Thorac Cardiovasc Surg.

